# Estimating the Smoking Ban Effects on Smoking Prevalence, Quitting and Cigarette Consumption in a Population Study of Apprentices in Italy

**DOI:** 10.3390/ijerph120809523

**Published:** 2015-08-13

**Authors:** Luca Pieroni, Giacomo Muzi, Augusto Quercia, Donatella Lanari, Carmen Rundo, Liliana Minelli, Luca Salmasi, Marco dell’Omo

**Affiliations:** 1 Department of Political Science, University of Perugia, via Pascoli 20, 06123 Perugia, Italy; E-Mail: luca.salmasi@unipg.it; 2 Unit of Occupational and Environmental Medicine, Department of Medicine, University of Perugia, Piazzale Lucio Severi, 1, 06132 Perugia, Italy; E-Mails: giacomo.muzi@unipg.it (G.M.); marco.dellomo@unipg.it (M.D.O.); 3 Dipartimento di Prevenzione, ASL Viterbo, Via Enrico Fermi 15, 01100 Viterbo, Italy; E-Mail: augusto.quercia@asl.vt.it; 4 Unit of Public Health, Department of Experimental Medicine, University of Perugia, Piazzale Lucio Severi 1, 06132 Perugia, Italy; E-Mails: dlanari@stat.unipg.it (D.L.); liliana.minelli@unipg.it (L.M.); 5 School of Public Health, University of Perugia, Piazzale Lucio Severi 1, 06132 Perugia, Italy; E-Mail: carmen.rundo@libero.it

**Keywords:** smoking bans, tobacco consumption, apprenticeship contracts, segmented regression

## Abstract

*Objectives*: We evaluated the effects of the Italian 2005 smoking ban in public places on the prevalence of smoking, quitting and cigarette consumption of young workers. *Data and Methods*: The dataset was obtained from non-computerized registers of medical examinations for a population of workers with apprenticeship contracts residing in the province of Viterbo, Italy, in the period 1996–2007. To estimate the effects of the ban, a segmented regression approach was used, exploiting the discontinuity introduced by the application of the law on apprentices’ smoking behavior. *Results*: It is estimated that the Italian smoking ban generally had no effect on smoking prevalence, quitting ratio, or cigarette consumption of apprentices. However, when the estimates were applied to subpopulations, significant effects were found: −1% in smoking prevalence, +2% in quitting, and −3% in smoking intensity of apprentices with at least a diploma.

## 1. Introduction

Clean Indoor Air Laws (CIALs) generally prohibit smoking in public places and require restaurants to provide non-smoking sections. The most restrictive of these laws also prohibit smoking in privately owned workplaces, in which the application of legalized restrictions will probably have a much greater effect on cigarette smoking due to the length of time they affect smokers.

Although in Europe CIALs are relatively recent, when they are applied to workplaces, the estimate of the effects on smoking outcomes appears to be important. For example, [1] estimated that employees working in a smoke-free workplace were more likely to make serious attempts to quit smoking and were also more likely to quit successfully than those who worked in places where smoking was allowed. Additionally, among smokers, employees in smoking-free workplaces consumed on average approximately three cigarettes per day less than those who worked in places with non-restrictive smoking policies. These results were confirmed in subsequent studies evaluating anti-smoking policies in Canada [2] and Finland [3].

In Italy, the CIAL implemented on 10 January 2005, imposed a wide-ranging smoking ban, prohibiting smoking in all public and private indoor areas open to the public, including workplace common areas, restaurants, cafés, and bars. The smoking ban had a high level of compliance [4] and significantly reduced both the numbers of smokers and the average number of cigarettes smoked by smokers in the Italian population [5]. Its introduction may, therefore, predict the positive effects of the ban on general health status, based on the known correlation between smoking habits and the prevalence of some types of cancer and cardiovascular diseases (see, for example, [6,7]).

However, there is little available knowledge regarding the specific effects of the application of the 2005 smoking ban in Italy on the smoking behavior of workers because there are no official surveys investigating this problem in workplaces. In this paper, we contribute to the debate of the effect about CIALs on workers’ smoking by exploring an administrative dataset of the population of apprentices in the province of Viterbo (Lazio region [Latium], central Italy) from 1996 to 2007. In Italy, apprentices are required to undergo medical examinations once a year, in which the aim is to prevent problems associated with workplace health and safety. An advantage of this approach is because apprenticeships are work contracts for young people, the effects of smoking can be examined in a young population which usually has a high prevalence of smoking and tobacco consumption compared to the general population of the same age. This situation is due to the fact that apprentices are mainly employed in unskilled work [8].

Our strategy exploits the application of the smoking ban by estimating the potential effects on smoking behavior through a segmented regression approach [9]. We take advantage of the assumption that other determinants of smoking may vary in time, although they would probably not have changed specifically in 2005.

We also extended our evaluation of the 2005 smoking ban in Italy on the smoking behavior of young workers to demographic characteristics. We based this evaluation on the literature that indicates that poorly educated people are less significantly affected by such bans, and age has a significant effect on participation rates and the consumption of cigarettes ([10,11,12]).

## 2. Sample and Variables of Interest

The dataset was obtained from the non-computerized records of the medical examinations of workers who reside in the province of Viterbo (Latium, Italy) for the population of apprentices from 1996 to 2007. 

Apprenticeship contracts were introduced in Italy with Law no. 25/1955, defined as contracts in which employers are responsible for providing or arranging vocational training in technical knowledge and professional qualifications. This type of training consists of a theoretical part, comprising complementary courses, which take place both inside and outside the firm and practical apprenticeship during working hours; all apprentices are also subjected to periodic medical examinations annually. A main characteristic of this type of work contract is that apprentices must be between 15 and 26 years old. However, with the regional application of the labor market reform in Italy (the so-called “Biagi Law” no. 30/2003), the upper age threshold was increased to 29 years of age in 2006 and 2007. For this reason, we decided to exclude from our sample individuals aged 27 or older to guarantee that our results are not driven by the variation in sample composition but are solely determined by the Italian smoking ban. 

During apprentices’ medical examinations, a questionnaire was administered by a trained healthcare provider. Because medical examination is a pre-requisite to be employed in a variety of business activities, industry, crafts and agriculture and commercial sectors, we registered full compliance. Information about apprentices is self-reported and concerns health status, smoking habits, consumption of alcohol, weight, physical activities, individual demographic characteristics and the sector in which apprentices are employed. Particularly, smoking habit was assessed by the following questions: (i) did you smoke at least 100 cigarettes in your life (*No, never smoker; Yes, smoker*); (ii) have you smoked regularly during the last year (if, *Yes*)? (*No, former smoker; Yes, current smoker)*; (iii) how many cigarettes do you smoke on average per day? The sample was comprised of 8291 individuals.

We distinguished current smokers (4287 workers), former smokers (265), and never smokers (3739) to obtain outcome responses in terms of prevalence and intensity of smoking and quitting ratio (*i.e.*, the prevalence of former smokers among ever smokers). 

We also used the Everyday Life Aspects survey (ELA), conducted by the Italian Institute of Statistics (ISTAT), which includes a representative cross-section of the Italian population and the demographics, social characteristics, and health of 20,000 households per year, corresponding to approximately 50,000 individual records/year. The ELA survey allows us to obtain representative statistics at the regional level because we cannot use data at the level of the province of Viterbo. Moreover, the ELA survey allows us to compare the smoking habits of apprentices because it collects comparable information with those obtained by the questionnaires administered to apprentices regarding gender, occupation, education, age and other demographic characteristics and detailed information on smoking habits (*i.e.*, if the respondent is a smoker, what product she uses and the number of cigarettes smoked). 

### Methods

In our empirical analysis, we first describe the smoking habits of our population of apprentices compared with those of a representative sample drawn from the general population of the Latium region. We selected a sample of individuals of the same age as our apprentices in the years 2001–2007 (except for 2004, when the survey was not conducted) from the ELA survey. Then, we calculated the smoking prevalence for the two samples in the available years (*i.e.*, 2001–2007, excluding 2004) and used a pairwise t-test to compare them. 

Then, to estimate the effect of the Italian smoking ban on apprentices’ smoking behaviors, we used the segmented regression approach recently adopted in the epidemiological field by [9,13]. The empirical model is specified as:

(1) $$y_{it}=\alpha_{1}+\alpha_{2}SB_{t}+\alpha_{3}Trend_{t}+\alpha_{4}{\left(Trend\text{*}SB\right)}_{t}+\sum_{j=5}^{J}\alpha_{j}X_{tij}$$

The dependent variables *y* are three outcome measures of smoking behaviors: (i) smoking prevalence (dichotomous variable that takes the value 1 if the respondent is a smoker and 0 otherwise); (ii) quit ratio (dichotomous variable that takes the value 1 if the respondent quit smoking and 0 otherwise); and (iii) smoking intensity (dichotomous variable that takes the value 1 if the respondent smokes more than 10 cigarettes per day and 0 otherwise). The coefficient α_2_ measures the immediate— short-term—impact of the smoking ban (SB) on outcomes, α_3_ estimates the presence of a linear trend in the outcomes of interest, and α_4_ estimates variations in the slope of the linear trend after the smoking ban came into effect. The coefficient of interest in our analysis is α_2_, which measures the impact of the smoking ban on smoking behaviors of the apprentices after the smoking ban was established. The inclusion of a linear trend and the interaction between the linear trend and SB ensure that the effect estimated by α_2_ is not affected by other macro-economic factors that may have happened simultaneously with the smoking ban introduction (*i.e.*, variations in cigarette prices or taxes). The estimation method used is the linear probability model (LPM) estimated through standard OLS. In this model, we do not include observations from ELA, but estimate the effect of the Italian smoking ban directly on apprentices.

We also included covariates, which control for the heterogeneity of respondents’ gender (man/woman), age (classes: <19, 19–23, >23), occupation (blue-collar/white collar), education (primary, diploma, or higher), and age at onset of smoking (classes: <15, 15–20, >20). We also included workplace location to account for possible geographical differences in smoking behavior. However, to examine heterogeneous responses in smoking habit variations after the ban came into effect, we focused our estimates on the effect of the ban in several subpopulations, divided by gender, education, and age.

## 3. Results

Table 1 lists the descriptive statistics for the socio-demographic variables included in our dataset according to smoking status. The percentage of women is clearly higher among former and never-smoker groups, whereas the gender proportions are balanced among current smokers. According to age, former smokers were generally older than current and never smokers. Smokers were more prevalent among individuals with a primary education, whereas those with diplomas or higher education were more likely to be never- and former-smokers. According to occupation, the percentage of current smokers was higher among blue-collar workers. We also showed that the average number of cigarettes smoked per day by current smokers and the age at which current- and former-smokers started to smoke. We noted that almost 70% of the sample smoked less than 10 cigarettes per day, 30% of individuals smoked between 10 and 20, and a very small number of individuals reported smoking more than 20 cigarettes per day. 

**Table 1:** Descriptive statistics.

Variable	Modality	Current Smokers	Former-Smokers	Never-Smokers
Gender	Women	0.49	0.63	0.56
	Men	0.51	0.37	0.44
Age class	<19	0.12	0.03	0.19
	19–23	0.65	0.58	0.61
	>23	0.23	0.39	0.20
Education	Diploma or higher	0.46	0.62	0.53
	Primary school	0.54	0.38	0.47
Occupation	White collar	0.67	0.78	0.74
	Blue collar	0.33	0.22	0.26
No. cigarettes/day	0	0.00	1.00	1.00
	< 5	0.17	-	-
	5–10	0.52	-	-
	11–20	0.30	-	-
	21–30	0.01	-	-
	31–40	0.00	-	-
Age at onset of smoking habit	<15	0.20	0.28	-
	15–20	0.75	0.64	-
	>20	0.05	0.08	-
Years	1996	0.03	0.02	0.04
	1997	0.07	0.03	0.08
	1998	0.12	0.08	0.11
	1999	0.14	0.08	0.13
	2000	0.14	0.15	0.13
	2001	0.06	0.08	0.06
	2002	0.07	0.09	0.07
	2003	0.07	0.08	0.07
	2004	0.08	0.10	0.08
	2005	0.08	0.13	0.09
	2006	0.06	0.07	0.07
	2007	0.08	0.08	0.07
Number of observations		*4287*	*265*	*3739*

**Figure 1:**
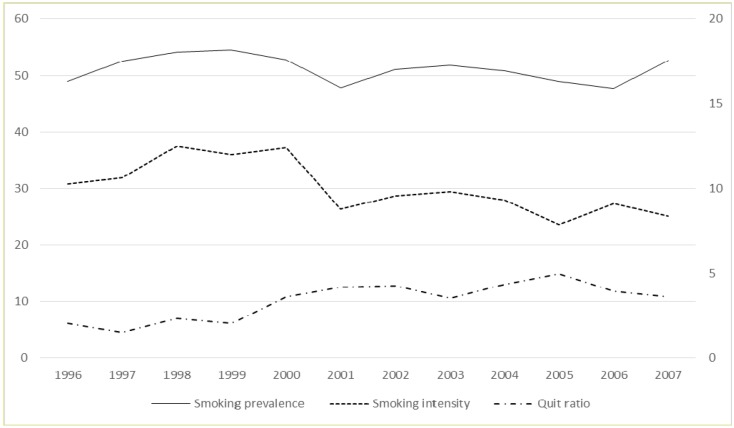
Trends in outcome variables 1996–2007. Smoking prevalence (individuals who currently smoke) and smoking intensity (individuals who smoke more than 10 cigarettes per day) on left axes, quit ratio (ratio between former smokers and ever smokers) on right axes.

Figure 1 shows the trends in the outcomes of interest. The solid, dash and dash-dot lines represent, respectively, smoking prevalence (% of smokers), smoking intensity (% of people smoking more than 10 cigarettes per day) and quit ratio (ratio between quitters and ever-smokers). In addition to the quit ratio that seems to present a slightly increasing trend over time, the other two outcomes do not highlight evident trends over time but instead show variability over time, which supports the use of the segmented regression approach to more flexibly model trends’ variations over time.

Table 2 shows comparisons of smoking prevalence of apprentices in the province of Viterbo with the population of Latium. The percentage of smokers among the apprentice population is approximately 50% per year. This proportion decreases to almost 25% compared to the Latium population of individuals of the same age. This finding indicates that the apprentices’ smoking prevalence is almost double that of the regional population. The third column of Table 2 lists the differences in smoking prevalence and the results of a pairwise t-test. As expected, the *p*-values indicated significant differences between the samples. Given the high level of smoking participation by apprentices, documented in Table 2, we expect substantial beneficial effects on this particular group from the introduction of the Italian smoking ban due to the increased individual costs of smoking, which identify the effort and time that smokers have to face to reach places where smoking is allowed. 

**Table 2:** Smoking prevalence of apprentices compared with the population of the Latium region (2001–2007), stratified by age.

Year	Apprentices	Population	Difference	t-Test (*p*-Value)
2001	0.48	0.25	−0.23	<0.001
2002	0.51	0.26	−0.25	<0.001
2003	0.52	0.23	−0.29	<0.001
2004	0.51	-	-	
2005	0.49	0.22	−0.27	<0.001
2006	0.48	0.23	−0.25	<0.001
2007	0.53	0.22	−0.31	<0.001
Total	*0.52*	*0.24*	−*0.28*	*<0.001*

Note: Calculation of smoking prevalence does not include 2004 (source not available).

Table 3 lists the estimated coefficients of the effect of the 2005 Italian SB on smoking habit outcomes. The estimated coefficient associated with the SB shows that no effect was induced in apprentices in terms of smoking prevalence, quit ratio, or smoking intensity.

**Table 3:** Effect of 2005 smoking ban on apprentices’ smoking prevalence, quit ratio, and smoking intensity.

Variables	Smoking Prevalence	Quit Ratio	Smoking Intensity
Smoking ban	0.081	0.131	−0.171
	(0.058)	(0.106)	(0.165)
Trend	0.001	0.002	−0.003
	(0.001)	(0.002)	(0.004)
Smoking trend X Trend	−0.009 *	−0.013	0.014
	(0.005)	(0.010)	(0.015)
Women *(Ref.)*			
Men	−0.011	−0.015 **	0.154 ***
	(0.007)	(0.008)	(0.015)
*Diploma or more (Ref.)*			
Primary education	−0.013	−0.015 **	0.094 ***
	(0.007)	(0.008)	(0.015)
*Age <19 (Ref.)*			
Age 19–23	0.011 ***	0.034 ***	0.225 ***
	(0.004)	(0.008)	(0.021)
Age >23	0.031 ***	0.072 ***	0.303 ***
	(0.006)	(0.012)	(0.025)
*White-collar workers (Ref.)*			
Blue-collar workers	−0.004	−0.006	0.075 ***
	(0.005)	(0.010)	(0.020)
*Age at start <15 (Ref.)*			
Age at start 15–20	−0.056 ***	−0.048 ***	−0.188 ***
	(0.011)	(0.010)	(0.018)
Age start >20	0.021	−0.023	−0.329 ***
	(0.020)	(0.022)	(0.029)
Constant	−0.005	0.085 ***	0.089 **
	(0.009)	(0.020)	(0.037)
Observations	8185	4447	4447
R-squared	0.04	0.02	0.10
Adj. R-squared	0.04	0.02	0.10

Notes: In this table, we show the marginal effects estimated through LPM of SB, linear trend, linear trend X SB and other covariates included in our model already described in previous sections on three outcomes that measure smoking behaviors (*i.e.*, smoking participation, intensity and quit ratio). Standard errors in brackets. Significant levels: *** *p* < 0.01, ** *p* < 0.05, * *p* < 0.1.

The same result appeared when we tested for a change in the slope of smoking habit trends, except in the outcome of smoking prevalence, which estimated a reduction of one percentage point at a significant level of 10%. This result led us to examine whether the ban had affected the smoking prevalence of specific groups. We shall return to this point below.

The estimated parameters for some socio-demographic variables confirmed the results already shown by the descriptive statistics in Table 1. Particularly, men and poorly educated individuals were less likely to quit smoking by two percentage points compared to women and more highly educated individuals, respectively, and they smoked a higher number of cigarettes. Instead, white-collar workers showed the same propensity to quit as blue collar workers, although the latter tended to smoke more (*i.e.*, 0.07 percentage points). 

Although smoking prevalence and intensity of cigarette consumption increased with age, the quit ratio also increased significantly for apprentices aged > 23. Lastly, apprentices who started smoking before the age of 15 were less likely to quit than those who started between 15 and 20, but no significant differences were found with respect to those who had started after the age of 20. Instead, in terms of smoking intensity, apprentices who started after 15 smoked fewer cigarettes.

Table 4 lists the estimates of the SB effect according to sample subpopulation. It should be noted that, although no significant effects were found according to the number of cigarettes smoked per day, among subjects with diplomas or higher education, there was a significant reduction of 1% in smoking prevalence and an increase of 2% in the quit ratio, whereas the reduction of 3% in smoking intensity was significant at the 10% significance level.

**Table 4:** Effect of 2005 smoking ban on apprentices’ smoking prevalence quit ratio and smoking intensity, stratified by socio-demographic status.

Variables	Women	Men	Diploma or Higher	Primary Education	Age <18	Age 19–23	Age >23
*Smoking prevalence*							
Smoking ban	0.094	0.045	0.124	−0.022	−0.083	0.034	0.185
	(0.073)	(0.095)	(0.073)	(0.093)	(0.064)	(0.070)	(0.124)
*Smoking prevalence*							
Trend	0.001	−0.001	0.001	0.001	−0.001	0.002 **	−0.003
	(0.001)	(0.002)	(0.002)	(0.001)	(0.001)	(0.001)	(0.004)
Smoking ban X Trend	−0.013	−0.003	−0.015 **	0.001	0.015	−0.003	−0.014
	(0.007)	(0.009)	(0.005)	(0.009)	(0.006)	(0.006)	(0.011)
Constant	−0.021	0.014	0.014	−0.015	0.031	0.001	0.035
	(0.012)	(0.014)	(0.025)	(0.010)	(0.025)	(0.009)	(0.026)
Observations	4358	3827	4061	4124	1231	5119	1835
R-squared	0.05	0.04	0.06	0.03	0.01	0.04	0.04
Adj. R-squared	0.05	0.03	0.06	0.03	0.01	0.03	0.03
*Quit ratio*							
Smoking ban	0.17	0.03	0.22	−0.05	−0.21	0.03	0.31
	(0.135)	(0.169)	(0.136)	(0.166)	(0.151)	(0.126)	(0.215)
Trend	0.00	−0.00	0.00	0.00	−0.00	0.01 *	−0.00
	(0.003)	(0.003)	(0.004)	(0.002)	(0.003)	(0.003)	(0.007)
Smoking x Trend	−0.02	−0.00	0.02 **	0.00	0.02	−0.01	−0.03
	(0.012)	(0.015)	(0.011)	(0.015)	(0.015)	(0.012)	(0.020)
Constant	0.06 **	0.07 **	0.16 ***	0.03 *	0.09	0.10 ***	0.17 ***
	(0.029)	(0.029)	(0.063)	(0.020)	(0.062)	(0.024)	(0.056)
Observations	2237	2210	2086	2361	518	2849	1080
R-squared	0.02	0.03	0.02	0.02	0.01	0.02	0.01
Adj. R-squared	0.01	0.02	0.02	0.01	−0.01	0.01	0.00
*Smoking intensity*							
Smoking ban	−0.08	−0.39	0.37 *	0.20	0.63	0.05	−0.49 *
	(0.188)	(0.324)	(0.198)	(0.294)	(0.564)	(0.227)	(0.278)
Trend	−0.00	0.00	0.01	−0.01	0.01	−0.01 *	0.02 **
	(0.005)	(0.006)	(0.006)	(0.006)	(0.010)	(0.005)	(0.010)
Smoking ban X Trend	0.01	0.03	−0.03 *	−0.02	−0.06	−0.00	0.03
	(0.018)	(0.030)	(0.019)	(0.027)	(0.050)	(0.021)	(0.027)
Constant	0.15 ***	0.21 ***	0.16 *	0.23 ***	0.23 *	0.37 ***	0.19 **
	(0.049)	(0.057)	(0.091)	(0.046)	(0.116)	(0.041)	(0.084)
Observations	2237	2210	2086	2361	518	2849	1080
R-squared	0.07	0.07	0.09	0.09	0.06	0.10	0.13
Adj. R-squared	0.06	0.06	0.09	0.09	0.04	0.10	0.12

Notes: In this table, we show the marginal effects estimated through LPM of SB, linear trend, linear trend X SB on three outcomes that measure smoking behaviors (*i.e.*, smoking participation, intensity and quit ratio) for selected subgroups of the population (*i.e.*, men and women, respondents with diploma or higher education and those with primary education, and by age groups <18, 19–23 and >23). Other covariates previously described were consistently included in the estimation of LPM but not reported in the table for the sake of simplicity. Standard errors in brackets. Significant levels: *** *p* < 0.01, ** *p* < 0.05, * *p* < 0.1.

## 4. Discussion

We found that apprentices’ smoking behavior was on average unaffected by the SB in Italy. The reductions in smoking prevalence and increases in quitting smoking were estimated only for apprentices with higher education. Significant determinants of smoking outcomes were gender, age, education, working status, and age at onset of smoking. 

A reduction in the prevalence of smoking and tobacco consumption was noted after the 2005 SB among Italian smokers. Observational studies have unambiguously shown that smoking habits and exposure were probably influenced by the ban in public places ([12,13,14]). Although there are no direct investigations on the influence of the ban on workers (and passive smoking) in Italy, it is probable that the law led to a substantial reduction in the prevalence of smoking and tobacco consumption in workplaces, as many American empirical reports suggest ([15,16,17]). Although laws affecting public places are likely to affect average smokers for a little more than a few hours a week, laws also affecting workplaces may regulate smokers' behavior for 40 or more hours a week. 

However, the potential for smoking restrictions to affect smoking by reducing the number of opportunities appears to lose influence in the case of apprentices. This remarkable difference highlights their specific case; they all have a large propensity to smoke, are young, have a lower education level, are mainly employed as blue-collar workers, and start smoking early. Our estimates controlling for confounding variables of the effects of the ban on smoking and tobacco consumption match the results found by [11] of “no effect”. The above authors examined the short-term effects of the introduction of the public SB in Germany in 2007–2008 and found that it did not change average smoking behavior within the population, although substantial differences emerged among socio-demographic groups. In our sample, women were less likely to smoke than men, and their smoking intensity was lower; these results also match those found by [18].

When we analyzed apprentices’ smoking outcomes by age class, we noted that smoking prevalence and consumption and quit ratio increased for individuals aged between 23 and 26 years. As argued by [10], the differential effect of the SB by age group may be due to differences in *initial smoking prevalence*. Smoking is indeed less than 47% for workers under 19 but well above 50% for those aged 19–23 years (52%), and, overall, the prevalence of smoking increased in the latter age class (23–26 years) to 54%.

Additionally, apprentices who started smoking before the age of 15 were less likely to quit and smoke more cigarettes. The probability that cigarette quitting and consumption is positively influenced by the length of the smoking habit, generally used as a measure of addiction, is well-known in the health policy literature (e.g., [19,20]). Suranovic *et al.* (1999) and Jones (1999) [21,22] formally theorized the underlying mechanism based on the trade-off between expected benefits and the fixed costs of quitting associated with the effects of nicotine dependence, which increases with duration. Particularly, when a subject with a previous lengthy smoking habit attempts to quit smoking (or to cut down greatly on the number of cigarettes smoked per day), a negative utility effect occurs because of the withdrawal effect.

As this work focused on the average effect on smoking behavior, variations in the probability of smoking may have limited implications because they do not refer to socio-demographic groups. When we examined the difference between these groups, we found that the SB significantly affected apprentice smokers with differing levels of education. The shock of this anti-smoking regulation induced a reduction in the percentage of more highly educated smokers and increased the probability of quitting. The literature on addiction shows that more highly educated smokers not only have lower levels of cigarette consumption but also a higher propensity to quit ([23,24]). 

The estimates produced in this paper were obtained through a segmented regression approach. However, because a variation in apprenticeship regulations—which allowed employers to hire apprentices until the age of 29—occurred in the same period of the Italian SB (*i.e.*, Law 30/03) and came into force in April 2007 in Lazio, we decided to exclude from our sample individuals aged 27 or more. This exclusion guarantees that our results cannot be affected by the variation in sample composition but solely by the Italian smoking ban. 

It should be stressed that our paper has some limitations although it uses an unexploited dataset for Italy. The questionnaire was administered by a trained healthcare provider and registers self-reported information about apprentices. This approach implies that the quality of data for some variables is not biochemically verified. However, by focusing on smoking behaviors, a wide participation in smoking of apprentices should exclude significant underreporting of smoking habits. Additionally, although we have no information about the type of sectors in which the apprentice will be employed, this limitation should not affect our results because the statistical distribution of apprentices interviewed are representative of the entire economy. 

## 5. Conclusions

In this paper, we show that the apprentices had a reduced prevalence of smoking and tobacco consumption if they were educated and that smokers with an education level of at least a diploma had an increased propensity to quit, although we must be cautious in concluding about the strength of the determinants that led to this result. In fact, as argued by [25], educated individuals are probably more aware of the health risks associated with smoking and are more prompt in responding to health improvements induced by the SB at both individual and collective levels.

Although the magnitude of the effects are small and they may therefore only be of limited clinical importance, they support previous study findings that workplace smoking bans may be an effective tool to improve worker health among more educated and less addicted smokers. This conclusion implies that additional public health action or other interventions may be needed to effectively reach less educated or more addicted smokers.
